# Concepts in Light Microscopy of Viruses

**DOI:** 10.3390/v10040202

**Published:** 2018-04-18

**Authors:** Robert Witte, Vardan Andriasyan, Fanny Georgi, Artur Yakimovich, Urs F. Greber

**Affiliations:** 1 Department of Molecular Life Sciences, University of Zurich, Winterthurerstrasse 190, CH-8057 Zurich, Switzerland; robert.witte@uzh.ch (R.W.); vardan.andriasyan@uzh.ch (V.A.); fanny.georgi@uzh.ch (F.G.); 2 MRC Laboratory for Molecular Cell Biology, University College London, Gower St., London WC1E 6BT, UK; a.yakimovich@ucl.ac.uk

**Keywords:** light microscopy, fluorescence microscopy, immunofluorescence microscopy, virus labeling, super-resolution, live imaging, image analysis, data analysis, high-throughput screening, modeling, simulation, computing, quantitative microscopy, fluorescent virions, microscopy, trafficking, membrane traffic, intracellular transport, machine learning, virus infection, DNA virus, RNA virus, enveloped virus, nonenveloped virus, cell biology, virus entry, cytoskeleton, infection, receptor, internalization, innate immunity, virion uncoating, endocytosis, gene expression, gene therapy, adenovirus, herpesvirus, herpes simplex virus, influenza virus, hepatitis B virus, baculovirus, human immunodeficiency virus HIV, parvovirus, adeno-associated virus AAV, simian virus 40

## Abstract

Viruses threaten humans, livestock, and plants, and are difficult to combat. Imaging of viruses by light microscopy is key to uncover the nature of known and emerging viruses in the quest for finding new ways to treat viral disease and deepening the understanding of virus–host interactions. Here, we provide an overview of recent technology for imaging cells and viruses by light microscopy, in particular fluorescence microscopy in static and live-cell modes. The review lays out guidelines for how novel fluorescent chemical probes and proteins can be used in light microscopy to illuminate cells, and how they can be used to study virus infections. We discuss advantages and opportunities of confocal and multi-photon microscopy, selective plane illumination microscopy, and super-resolution microscopy. We emphasize the prevalent concepts in image processing and data analyses, and provide an outlook into label-free digital holographic microscopy for virus research.

## 1. Introduction

Viruses are wide-spread and occur in massive numbers in the biosphere. Within hosts, viruses evolve rapidly, and infect cells despite opposing innate and adaptive host immunity. Virus particles, so-called virions, transfer DNA or RNA genomes between cells and organisms, and cause infections. They give rise to morbidity and mortality, or persistent and latent infections. Viruses are tightly interconnected with cellular processes, are genetically highly adaptable and emerge unpredictably. This makes it difficult to combat them and to develop effective, fast-acting, and long-lasting anti-viral agents. Imaging viruses and cellular processes by light microscopy presents a major opportunity to enhance knowledge about virus infections, and find new angles for anti-viral interference. While electron microscopy (EM) images of heavy metal stained specimens have been developed in the 1930s, pioneering light microscopy experiments of viruses were initially only described in the 1980s [1,2,3].

Ever since the early days of microscopy, virus imaging has continued to inspire the development of new concepts in cell and virus biology. In recent years, research of viruses, cells, and organisms has been extended by the acquisition and the processing of big data and digitalization. Large data in experimental biology are obtained through large scale screening projects in perturbation experiments. For example, big data are used to interrogate the host and pathogen genetics, the metabolism of infected and uninfected cells, the flux of inorganic ions, the response of the immune system to restore homeostasis, or how infected cells die. Coincidentally, automated digital image analyses and data-based studies facilitate a transition from phenomenology to exploration of hypotheses. While big data correlations and large-scale imaging allow for the rapid identification of unexpected phenotypes, they also come at the cost of errors and artefacts, and require in-depth validations by follow-up studies for mechanistic interpretations.

Static and live cell imaging of infected cells have emerged as powerful approaches to validate big data. This is based on a long history of imaging in the natural sciences and distinguished by Nobel prizes, for example ultramicroscopy [4], phase contrast imaging [5], holography [6], electron microscopy and scanning tunneling microscopy [7], optical super-resolution microscopy [8], and cryo-electron microscopy [9].

The visualization of micrometer-sized bacteria by van Leeuwenhoek opened-up the fields of optical microscopy and microbiology in the 17th century [10]. The development of microscopes for the detection of nanometer-sized viral particles only occurred centuries later, in part due to physical limitations in optics. In 1873, Ernst Abbé postulated the diffraction limit for optical microscopes [11]. The Abbé limitation restricts the resolution of traditional optical microscopy to approximately 250 nm lateral and 700 nm axial resolution. Further advances in imaging systems harnessed shorter electromagnetic waves, such as X-rays and electrons. This paved the path to the first depictions of tobacco mosaic virus (TMV) in 1939 [12], the prediction of the structure of TMV by Rosalind E. Franklin [13], and the first high resolution structure of a virion, tomato bushy stunt virus [14]. 

While initial applications of microscopy in virology were predominantly EM-based, the fields of cell and developmental biology were strongly influenced by light microscopy. The conceptual merger of cell biology and virology was largely influenced by the work of Kai Simons, Ari Helenius and colleagues in the 1980s, and opened the doors for virology into light microscopy [1,2,3]. Over the last two decades, light microscopy techniques have experienced phenomenal improvements in sensitivity, functionality and resolution, including resolutions down to 1 nm, single-molecule detections, volumetric (3D) live-imaging, imaging over extended periods of days and weeks, and combinations thereof [15,16,17,18].

Here, we summarize the methodology for imaging virus infections of cells and virions by light microscopy, including the recent static and live cell fluorescence microscopy approaches and image analyses procedures. We also provide a view on emerging technologies. For a comprehensive overview of common imaging modalities see Table 1.

## 2. Probes and Labeling Strategies for Imaging of Viruses

Optical microscopy encompasses imaging modalities utilizing the spectrum of the visible light. This includes for example phase contrast microscopy [22,23], or digital holographic microscopy [24,25,26], see Section 6.2. The most common procedures in cell biology are however designed to detect one or several fluorophores by fluorescence microscopy. Small chemicals, or transgenic proteins expressed in cells of interest are the most widely used fluorophores. Alternative fluorescent tags comprise semiconductor particles, such as quantum dots (Q-dots) [27,28], or reactive chemical groups allowing for covalent attachment of a fluorophore or another chemical using click chemistry [29,30]. The choice of label depends on the imaging method, for example imaging of fixed or live cells. Increasing capabilities in spatial resolution increase the importance of a small size of the tags. In typical immunofluorescence, for example, primary and secondary antibodies have a size of 10–15 nm each, resulting in significant discrepancy in the location of target and tag in super-resolution microscopy applications [31]. Smaller labels, such as Q-dots (discussed in Section 2.2), nanobodies (discussed in Section 2.3 and [32]) or aptamers (see review [33]) may help to resolve this issue. Here, we give an overview about different types of fluorophores in major applications, and discuss promising new dyes and procedures.

### 2.1. Labeling of Chemically Fixed Specimens with Single Molecule Sensitivity

Immunofluorescence (IF) or fluorescence *in situ* hybridization (FISH) have been used in life sciences for decades [34,35]. Both methods require the fixation and exposure of the target protein, lipid, or nucleic acid. Chemically fixed cells are relatively stable, and provide access to intracellular structures depending on the fixation and extraction procedure, albeit at the cost of compromising the integrity of the native cell [36,37]. Fixed and permeabilized cells are accessible to antibodies in IF analyses, or to oligonucleotides in FISH stainings. Fixed samples are incompatible with live imaging. Yet, they allow photon sampling over extended acquisition times, and hence the visualization of dim signals and events occurring too fast for live imaging.

Classically, it has been difficult to obtain sufficiently strong signals from single molecules with classical fluorescence or confocal microscopy. In recent years, more elaborate staining methods have been developed, which have sufficient sensitivity for single molecule detection by traditional confocal or wide-field microscopes. A first approach was single molecule FISH (smFISH), which made single molecule detection possible due to multiple specific short probes that can be used on a particular nucleic acid target which is hundreds of nucleotides in length [38,39]. This approach has been used, for example, to visualize viral RNAs of Influenza A virus (IAV) or Hepatitis C virus (HCV) in infected cells [40,41,42]. A slightly different approach is the so-called branched DNA (bDNA) technique, which generates a multi-layered scaffold for fluorophore binding and thereby drastically increases the number of probes bound near the target [43,44,45]. Both approaches have been combined to generate several scaffolds per target molecule [46,47], and thereby result in bDNA foci depicting single target molecules at high sensitivity and low background. Currently, commercial assays available include ViewRNA ISH Cell Assays (ThermoFisher Scientific, Waltham, MA, USA) and RNAscope (Advanced Cell Diagnostics, ACD, Newark, CA, USA). Although these assays require more time and are more expensive than traditional FISH, they effectively detect different viruses with single molecule sensitivity, for example Zika virus [48], HCV [49], Hepatitis B virus (HBV) [50], or human papilloma virus (HPV) [51].

A different single molecule imaging approach is points accumulation for imaging in nanoscale topography (PAINT). PAINT is based on a similar idea as direct stochastic optical reconstruction microscopy (dSTORM), and uses freely diffusible tags to achieve target blinking. The original implementation of PAINT achieved precisions of 25 nm in a system that transiently labeled lipids via hydrophobic interactions with a fluorescently marked transferrin [52]. The system was simplified by the use of DNA probes to achieve programmable interaction kinetics and high specificity of oligonucleotide interactions [53]. Current implementations achieve 3D super-resolution at 10 nm [54], and 2D resolution down to 1 nm [55,56], and have been used in quantitative super-resolution imaging [57].

While the sensitivity of single molecule techniques was improved drastically, the limited accessibility of the target imposes major restrictions. In virology, this has been noticed in the 1990’s, when conventional FISH revealed the incoming adenovirus (AdV) DNA genomes predominantly in the cell nucleus but not effectively in the cytoplasm [58,59]. One solution to circumvent this issue is the direct labeling of the viral genome with a probe that acts as a reaction partner for the attachment of a reporter molecule through click chemistry. Click chemistry describes a class of modular, biocompatible chemical reactions that result in the covalent attachment of a reporter molecule, such as a fluorophore to a biomolecule [60]. The prototypic implementation of click chemistry has been copper-catalyzed azide-alkyne cycloaddition, which combines fast reaction kinetics, high yields, and high accuracy [61].

One powerful application of click chemistry in virology has been the use of nucleoside analogues containing an alkyne group. For example, ethynyl-modified nucleosides are cell-permeable, can be incorporated into viral genomes, and thereby provide the reactive groups for azide-modified probes upon cell fixation and permeabilization. This technique has recently led to the notion that incoming adenoviral or herpes viral DNA is not only imported into the nucleus but also misdelivered to the cytoplasm [62,63,64]. Furthermore, this approach has enabled the tracking of the incoming viral genome at single genome resolution [62,64,65], and the isolation of proteins and micro-RNAs interacting with the viral genome [66,67,68,69]. In recent years, live cell and live animal compatible click chemistry protocols have been developed that allow labeling of lipids, albeit at lower sensitivity than copper-cased alkyne-azide cycloaddition [70,71,72]. Besides modified nucleosides, several click chemistry compatible derivatives of amino acids, sugars, and lipids have been developed. l-azidohomoalanine, for example, was used to study eIFα phosphorylation during respiratory syncytial virus (RSV) infections [73], *N*-azidoacetylgalactosamine to test how glycoprotein modifications of paired immunoglobulin-like receptor α affected the binding of herpes simplex virus (HSV) 1 to cells [74], and azide-modified palmitic acid to study the impact of the retromer on vaccinia virus (VACV) egress from infected cells [75]. For an overview of currently available products, see [76].

### 2.2. Live Cell Imaging of Viruses Labeled with Organic Fluorophores and Quantum-Dots

Phase contrast microscopy was initially used to visualize viruses in live cells, specifically the budding process of IAV at the plasma membrane of cultured cells [77]. Soon after, a range of virus particles was tagged with fluorescent dyes, including IAV, AdV, adeno-associated virus (AAV), HPV, human immune-deficiency virus (HIV) and simian virus (SV) 40 [1,78,79,80,81,82]. This development represented an important advance for follow-up mechanistic studies of virions in cells. For reviews, see [15,18,78,83,84,85,86,87,88,89,90,91,92,93,94,95,96,97,98,99,100].

In addition to organic fluorophores, Q-dots have been used to study virus infections. Q-dots are nanometer-sized semiconductor crystals absorbing and emitting photons. Compared to organic fluorophores they have broader excitation spectra, superior photobleaching resistance, improved far-red absorption, and adjustable blinking properties [28]. These features make them well suited for demanding applications, such as 2-photon microscopy [101], fluorescence resonance energy transfer (FRET) [102,103], or imaging of objects at low signal-to–noise ratio (SNR) [104,105,106]. Q-dots per se cannot pass through the lipid bilayer and have no inherent affinity to the major classes of cellular macromolecules, proteins, sugars, nucleic acids and lipids. Labeling viral particles with Q-dots through streptavidin-biotin interactions, functionalization with oligonucleotides or direct encapsidation in the virion have been utilized in studies with RSV, AdV, and HIV, and allowed virion tracking during entry into cells [107,108,109,110,111]. Other approaches included transfection, electroporation, and microinjection of Q-dots [112], for example to map HIV-1 provirus integration loci [113].

The scope of live cell fluorescence imaging has been largely extended by use of nonpolar reagents, which are cell-permeable. A prototypic cell-permeable small molecular weight dye is Hoechst 33342, which has been used for DNA staining in live cells since the 1970s [114]. The Hoechst dyes absorb and emit light in the ultraviolet range, and are frequently used in live imaging, for example, to monitor DNA contents during the cell cycle, or viral replication compartments in the cytoplasm [62,115,116]. Other dyes that are frequently used in live cell imaging are DRAQ5 to stain DNA in the far-red excitation spectra [117], the mitochondrial dye chloromethyl-X-rosamine (MitoTracker) [118] and a series of lysosomotropic dyes for acidic vesicles [119].

A recent addition to live cell markers are silicon-rhodamine (SiR) dyes. SiR dyes are derived from the red-spectrum dye tetramethylrhodamine (TRITC), and give strong fluorescence signals, with high photostability and good cell permeability [120]. High affinity SiR-dyes were developed for actin and tubulin, called SiR-actin or SiR-tubulin, and are suitable for live cell imaging due to their high membrane permeability [121]. Similarly, SiR-Hoechst and SiR-lysosome resulted in DNA, and lysosome stainings at low toxicity [122,123]. The SiR-dyes were chemically modified to fine-tune the spectral properties of the fluorophores while retaining cell permeability [124]. In addition, azetidine substitution for the amino group of rhodamine resulted in the Janelia Fluor dyes with excellent fluorescent properties and cell permeability, useful in demanding applications, such as two-photon and super-resolution microscopy [122]. Similar to the substitution strategy in rhodamine, a method for tetrazine-based fluorophores has been described to generate a series of compounds with a wide range of emitted light and suitability for live visualization of mitochondria [125].

### 2.3. Transgenic Approaches for Live Cell Imaging of Viruses

Genetically encoded fluorophores can be expressed in cells, and their detection requires no additional staining or labeling procedures. Different protein-based fluorophores, such as the green fluorescent protein (GFP) or variants with different excitation/emission spectra can be genetically fused to proteins of interest and used to visualize different structures at the same time [126]. They may be expressed under constitutive or inducible promoters, or from an endogenous promoter to expression levels in a physiological range.

The prototypic fluorophore GFP was first isolated in 1962 [127]. It took more than three decades until it was utilized as a tag in live cells [128]. Transgenic fluorophores, such as GFP, or the more stable enhanced GFP (eGFP) [129] are well suited for live imaging. They provide a good SNR, and do not require extensive sample preparation [130]. GFP has initially been used as a generic reporter for infection, or an indicator of viral transcription or replication [91,94,131,132]. If fused with a cellular or viral protein, the fluorescence provides spatio-temporal information about the fusion protein [133,134,135,136]. This requires engineering of the viral genome, as shown with a variety of viruses, including AdV [135,137], VACV [116], HIV [138], IAV [139], and HSV [140].

The stable expression of fluorescent proteins also reveals the dynamics of virus–host assemblies. For example, the stepwise engagement of the ESCRT machinery in the budding process of HIV particles has been visualized by fluorescence microscopy of tagged env and gag proteins of HIV in conjunction with VPS4-GFP [141]. Another example is from viruses affecting the cell cycle [142,143]. This can be analyzed with the fluorescent ubiquitination-based cell cycle indicator (FUCCI) GFP/RFP reporter system. Here, Cdt1 and Geminin are genetically fused to red or green fluorescent proteins, which undergo cell cycle-dependent proteolysis. This allows live cell imaging at single cell resolution, which is suitable to study how pathogens affect the cell cycle [144,145,146,147,148]. The FUCCI system was recently extended by additional fluorophores, and together with other enhancements, this now resolves the different cell cycle stages even better [149,150].

Combinatorial multi-color GFP variants, also known as the brainbow cassette, comprise multiple floxed fluorogenic proteins, which can be randomly excised in cells expressing the Cre recombinase [151]. Originally implemented in transgenic mice for tracking of neurons, it was adapted to imaging of viruses. In case of HSV, brainbow gave rise to a variety of reporters distinguishing individual neurons in the brain [152]. Multiple fluorogenic genes have also been designed for AAV [153]. The approach can be used to assess virus replication, competition, spreading, or the arrangement of infected cells in tissue [154].

Genetically encoded fluorescent proteins have also been reported for tagging of viral DNA in the context of the Tet-repressor (or Lac-repressor) and operator system, where the viral genome was engineered with Tet/Lac operator sequences that recruited the soluble Tet/Lac-repressor-GFP fusion protein [155,156,157,158]. While the approach is potentially useful to record the dynamics of newly replicated viral DNA, it has severe limitations for tracking the incoming viral genome, even if dozens of Tet/Lac-operator sequences are engineered into the reporter virus [159]. Limitations arise due to the low SNR, the noncovalent interaction between the tet-repressor and the viral DNA, and most importantly, the uncontrollable accessibility of operator sequences for the soluble Tet/Lac-repressor, for example due to packaging of the viral DNA into capsids.

An alternative approach for irreversibly tagging cell proteins under physiological conditions is by enzymatic labeling. Enzymes which covalently attach a cell-permeable fluorophore can be genetically fused with a protein of interest, expressed in cells and used to transfer the fluorophore to specific protein sites in the immediate vicinity of the catalyst. An early example of enzymatic in situ labeling was SNAP [160], later enhanced by variations including CLIP and HALO tags [161,162]. For a comprehensive overview of protein tagging techniques both in cell biology and virology see [163,164,165]. Light microscopy can also control local protein–protein interactions, for example by using genetically encoded light-inducible protein dimerization [166,167]. A big advantage here is the high speed of dimerization under in situ conditions [166]. 

As of now, it is not possible to dynamically image incoming viral DNA in live cells prior to the onset of viral transcription or replication, largely due to the difficulty to deliver suitable fluorophores across the plasma membrane into cells [70,120]. Another limitation is the relatively high amount of effort for generating transgenic viruses or stable cell lines, particularly if expression levels of the fluorescent protein are intended to remain in the physiological range. One approach to label proteins is by expressing plasmid-encoded proteins of interest fused to a nanobody or a fluorescent protein [168,169]. Using nanobodies directed against known epitopes allows fast generation of probes for live imaging at high sensitivity [170]. The high stability and specificity, together with the small size of nanobodies of about 4 nm, reduce the likelihood of interference with the function of the tagged protein. Importantly, the small size of the nanobody-tag also enhances the accuracy of target protein localization in super-resolution modalities [32]. 

For the visualization of nucleic acids by oligonucleotide probes, the CRISPR/Cas technology is increasingly harnessed as it readily allows targeting of proteins to specific nucleic acid sequences in a nucleotide specific manner [171,172]. Fluorescent versions of the CRISPR/Cas system have been designed that allow the visualization of host nucleic acids in live cells, such as telomeres [173], RNA [174], or HIV proviral DNA integrated into host chromosomes [175].

## 3. Diffraction Limited Microscopy

The Abbé diffraction limit illustrates the practical limitation of optical microscopes, achieving approximately 250 nm of lateral resolution. This is insufficient to resolve nano-structures, such as receptors, virus particles, or cytoskeletal filaments. Yet, diffraction limited microscopy, including widefield transmission or phase contrast microscopy, high-content imaging, confocal microscopy or selective plane illumination microscopy (SPIM) are generally more sensitive than super-resolution fluorescence microscopy, since the latter can induce rapid bleaching of the fluorophores or discard a significant fraction of the emitted photons, which limits the SNR. In addition, several super-resolution approaches depend on an excess of light and therefore induce significant phototoxicity in live samples. Live-cell imaging and 3D image acquisition can be easier implemented by diffraction-limited microscopy than super-resolution microscopy due to higher acquisition speeds and reduced phototoxicity. The operation requirements and the relative ease of accessibility of common imaging modalities are highlighted in Table 1 and Table 2. In particular, Table 2 illustrates why diffraction-limited microscopy is broadly used.

### 3.1. Confocal Microscopy

Confocal microscopy has been widely used for imaging of virus infections at the cellular level [176]. The concept of the confocal microscope was initially inspired by the slit lamp used in ophthalmology [177]. The first confocal scanning microscope was built in the 1950s [178]. The invention of laser technology led to the initial introduction of the confocal laser scanning microscope (CLSM) in 1971 [179]. Unlike widefield microscopy, CLSM utilizes a spatial slit, sometimes called a pinhole, to block out-of-focus light. Reflective and selectively permeable mirrors direct light to the sample and collect the emitted light from the sample in scanning mode. The highly focused laser spots can, however, induce phototoxicity and photobleaching in live or fixed samples. Spinning disk confocal microscopes (SDCM) somewhat circumvent this problem by probing the sample through multiple pinholes arranged on a Nipkow disk, which enables faster scanning and acquisition of live cell information. Furthermore, SDCM setups typically utilize high sensitivity cameras with better quantum efficiency (QE) compared to point scanners used in CLSM setups, thus enabling better visualization of dim viral particles. Yet, the benefit of reduced phototoxicity by SDCM comes at the cost of spatial resolution [99]. For a detailed comparison of different confocal microscopy techniques, we refer the reader to [180].

In recent years, manufacturers of confocal microscopes have developed systems that combine high-content image-based screening with spinning disk confocality. Examples of such systems include the IXM-C series from Molecular Devices [181], the Yokogawa CSU-X1 [182], Opera Phenix from Perkin Elmer [183], the IN Cell Analyzer 6000 from GE Healthcare [184], or further instruments from other suppliers [185]. The commercial systems differ in image quality and practicability, for example, the possibility of phase-contrast acquisition, light sources, image file formats, the feasibility to exchange the optical filters, or price. Future developments in high-content screening are ongoing, for example using correlative high-throughput light microscopy with targeted super-resolution acquisition [186].

### 3.2. Multi-Photon Imaging

Multiphoton microscopy, also known as two-photon microscopy or non-linear laser scanning microscopy was first introduced in 1931 [187]. It is diffraction-limited but holds significant advantages over conventional confocal microscopy and deconvolution procedures. Two-photon microscopy enables efficient three-dimensional optical sectioning of deep-tissues at distances of millimeters from the surface with low phototoxicity and photobleaching. It is well suited for imaging of living cells and tissues, such as brain slices, embryos, organs, and even small animals.

Unlike CLSM and SDCM, multiphoton microscopy does not use apertures for generating contrast and optical sectioning. It is based on the principle that simultaneous absorption of multiple rather low energy photons can induce a fluorophore to emit a higher energy photon in one quantum event. As the probability of such an event is very low, multiphoton microscopes typically employ high power impulse lasers. This results in a dumbbell-shaped point spread function, which leads to improved axial resolution and optical sectioning compared to single photon excitation (see also Section 5.1). Moreover, the excitation spectra are in the low energy infrared region, and therefore induce little cell death, even at extended imaging times. The superior penetration depth and low phototoxicity make multiphoton microscopy interesting for studying host–pathogen interactions intravitally, although logistic adjustments to match biosafety standards remain a challenge [188,189]. Furthermore, multiphoton microscopes allow for integrating additional imaging modalities, such as fluorescence recovery after photobleaching (FRAP) to measure diffusion kinetics of a fluorescent entity [190], fluorescence-lifetime imaging microscopy (FLIM) used for visualizing the lifetime of individual fluorophores [191], and coherent anti-Stokes Raman spectroscopy (CARS), which allows for label-free imaging based on vibrational signatures of biological samples [192,193].

### 3.3. Fluorescence Resonance Energy Transfer

Fluorescence resonance energy transfer or Förster resonance energy transfer (FRET) is a near-field interaction phenomenon between two light sensitive molecules. The phenomenon is based on non-radiative dipole–dipole coupling and occurs when an initially excited molecule, the donor chromophore, transfers energy to a nearby molecule, the acceptor chromophore. The efficiency of energy transfer exponentially declines with the distance between the donor and acceptor chromophores, making FRET a good indicator of molecular proximity in the low nm range [194,195]. The difference in donor and acceptor emission is the FRET efficiency, which can be quantified as a two-color ratio, and is commonly referred to as sensitized emission [196]. However, cross-talk between the chromophore emission spectra can represent a significant challenge, and requires extensive control experiments to extract the true FRET signal.

An alternative approach is to use acceptor photobleaching or donor dequenching, which measures FRET efficiency by the photobleaching of donor, depending on the proximity of the acceptor [197]. Yet, another approach is to reduce the crosstalk by coupling FRET with FLIM [191,198]. In this case, the efficiency of FRET is measured as the lifetime of the donor fluorescence, which depends on the proximity to the acceptor. In sum, FRET is widely applicable for exploring host–virus interactions [198,199,200].

### 3.4. TIRF Microscopy

Apart from challenges to improve spatial and axial resolution, the small size of viral particles poses a detection challenge with respect to SNR. As discussed above, the signal can be improved by increasing the QE or number of tags per virion. Another way to improve the SNR is to reduce the noise, e.g., by minimizing background fluorescence of the medium and the specimen [18,201]. The reduction of autofluorescence may also be achieved by changing the sample illumination from a straight beam (epi-illumination) to angled illumination or total internal reflection fluorescence (TIRF) [202]. In TIRF mode, most of the excitation light is reflected by the medium boundary when passing from a medium with a higher refractive index to a medium with lower refractive index (specimen). A portion of this light, the so-called evanescent wave illuminates the part of the specimen closest to the coverslip, about 100–200 nm [203]. This way the background from scattering, illumination of the out-of-focus fluorophores, and sample autofluorescence is avoided, and SNR increased up to 30-fold.

TIRF microscopy is a simple and efficient way of boosting detection sensitivity for small virions or virions labeled with low quantum yield dyes. Furthermore, TIRF microscopy is fully compatible with live cell imaging, making it well suited for imaging of fast moving intracellular and extracellular particles [99,204], or cell and tissue movements in morphogenesis of developing embryos [205]. In there, the remarkable SNR obtained by TIRF microscopy makes TIRF the method of choice for image acquisition of stacks to be used for localization in super-resolution microscopy.

### 3.5. Selective Plane Illumination Microscopy

In 1993, orthogonal plane fluorescence optical sectioning (OPFOS) [206] was first applied to biological samples [207]. A modern version of OPFOS is selective plane illumination microscopy (SPIM) or lightsheet imaging, pioneered by Huisken and Stelzer [208]. In contrast to epifluorescence imaging, only the observed plane of the specimen is illuminated perpendicular to the observation axis of a widefield microscope, typically a few hundred nanometers in thickness. Advances of SPIM include highly parallelized acquisitions, which make it suitable for long term imaging with high temporal resolution, as well as extremely low phototoxicity. In addition, light scattering effects, which reduce the light penetration depth, are reduced by pivoting of the light sheets. SPIM further improves light penetration of the tissue by imaging upon axial rotation of the sample resulting in multiple z-stacks. A full 3D model of the sample is then obtained by a computationally heavy alignment of the different image stacks. The 3D images can be further enhanced by image post-processing such as deconvolution [209]. So far, SPIM has been most successfully used for highly transparent samples or shallow cell layers, for example in embryogenesis [210,211].

Broad applications of SPIM in virology are awaited, but convenient commercial microscopes such as the ZEISS Z.1 (Carl Zeiss, Oberkochen, Germany) or the Leica TCS SP8 DLS (Leica Microsystems, Wetzlar, Germany) will likely facilitate broader applications. Wider application of SPIM may be enhanced also by tissue clearing protocols which turn the opaque brain tissue into a see-through structure. Several protocols and iterations have been published, such as CLARITY [212], PACT [213], or iDISCO [214]. For compiled features of tissue clearing procedures, see [215,216]. Recent extensions of clearing protocols now allow for an effective deblurring of organelles in tissues that have been difficult to image, or even clear whole-body specimens, expanding the functionality to non-neurological viruses [213,217].

An interesting trait of DISCO, specifically uDISCO, is the combination of tissue clearing with shrinking to allow easier handling and imaging of large sized organs [218]. With increased availability and simplicity of 3D acquisition and sample preparation protocols we expect an increase in SPIM and related 3D imaging of virus infections.

### 3.6. Expansion Microscopy

The reverse approach to shrinkage microscopy is expansion microscopy (ExM). It combines tissue clearing with 3D expansion of the sample. In ExM, the sample is embedded in a polymer, and upon rehydration allowed to expand in an isomorphic manner [219,220]. A single step ExM can achieve a linear expansion of approximately 4.5-fold and an effective resolution up to 50 nm [221], which can be increased by iterative ExM to 20× expansion and ~25 nm resolution [222]. So far, the system has been used for neurological samples, including virus induced pathological effects [223]. Other studies utilized it to observe cellular changes upon Influenza infection [224], and analysis of *Escherichia coli* bacteria [225]. To date, no study has used ExM to expand and visualize viral particles. In sum, ExM enhances the resolution of clustered objects, and is a rather easy to implement alternative to super-resolution microscopy.

## 4. Super-Resolution Microscopy

### 4.1. Super-Resolution Imaging

Super-resolution microscopy overcomes Abbé’s diffraction limit. It represents a key advance in optics for the life sciences, and has been awarded the Nobel Prize for chemistry in 2014 [8]. While geometrical super-resolution microscopy enhances the resolution of digital sensors, optical super-resolution overcomes the diffraction limit by optics [226]. Two distinct strategies have been developed—deterministic super-resolution and stochastic super-resolution. Stochastic approaches include stochastic optical reconstruction microscopy (STORM) [227,228] and photoactivated localization microscopy (PALM) [229], which uses the random variance of the label in multiple exposures, and therefrom calculates the true spatial location of the signal. Deterministic super-resolution enhances the non-linear excitations of fluorophores, and allows the determination of the true origin of the signal.

Examples for this approach are stimulated emission depletion (STED) [230,231,232], and structured illumination microscopy (SIM). While STED induces phototoxicity by the high-power laser pulse for quenching the of out of focus light, the phototoxicity is considerably lower than in stochastic imaging. STED is well-suited for super-resolution live-cell imaging [21,233]. For example, it has been employed to uncover that the incoming adenoviral DNA genomes are not solely delivered to the nucleus but are also misdelivered to the cytosol, and there give rise to innate immune activation [62,63,234]. STED was also used to document the entry of pseudotyped HIV particles [235], and to visualize HIV gag processing and lattice rearrangement during proteolytic virion maturation [17].

In SIM, multiple phase shifted images are acquired and overlaid to generate several interference patterns. By combining the information in these patterns with the image in Fourier space, a frequency function can enhance image resolution [236]. First-generation SIM microscopes achieved a lateral resolution of about 130 nm [237]. Recent implementations accomplished 50 nm resolution [238], while currently available commercial systems claim 20 nm resolution [239]. Advantages of SIM comprise relatively simple image acquisition procedures, and high signal to noise with a wide range of fluorophores. In fact, any fluorophore stable enough to endure multiple illuminations without major bleaching is compatible with SIM, including fluorescent dyes, such as Hoechst dyes, eGFP, Alexa-dye labeled antibodies, and combinations thereof [240]. Major drawbacks of SIM are the low speed of image acquisition and high phototoxicity [241]. Another drawback is the heavy load of computational analyses and image processing required in time-lapse series or z-dimension resolved imaging [242].

Modifications in the light path of the microscope have been proposed to address these issues. For example, instant structured illumination microscopy (iSIM) combines classical scanning confocal microscopy principles with multiplexed detectors capable of fast super-resolution image acquisition and processing [243,244]. iSIM is based on a sparse lattice pinhole array for local excitation and out-of-focus light rejections [245]. A second approach is called “Airyscan”. It is based on the classical scanning confocal microscopy setup, and is enhanced by multiplexed detectors. For further details, see below and [246]. The simple usage of the commercially available SIM instruments has inspired many virological applications, such as tracking of HIV particles [247], lytic granules [248], or the degradation of nucleoporins in IAV infection [249]. Notably, SIM is under continuous improvement, and recent advances reported SIM super-resolution imaging at 10 Hz [250].

### 4.2. Image Scanning Microscopy

Sufficiently small pinholes in standard confocal microscopes can theoretically achieve super-resolution. In practice, however, this would result in unfeasible low signal. Image scanning microscopy (ISM) circumvents this issue by combining confocal imaging with wide-field charge-coupled device cameras and performing pixel reassignment to achieve approximately 2-fold resolution improvements [251]. While easy to implement, limitations in camera design have resulted in prohibitively slow acquisition speed. Significant speed improvements were achieved by acquisition parallelization with a spinning disc confocal setup [245,252,253]. The Zeiss “Airyscan” confocal microscopes are equipped with multiplexed detectors arrayed in a way to allow for either high sensitivity, scanning speed or super-resolution imaging [16,254,255]. While requiring great precision in the detector alignment, the image acquisition process is rather simple, and effectively provides super-resolution quality at similar effort as in standard confocal acquisition. To date, ISM provides only moderate improvements in resolution. However merging ISM with 2-photon microscopy has achieved super-resolution at 250 µm tissue depth [256], or yielded high speed image acquisition at 30 Hz in 100 µm tissue depth with enhanced resolution and contrast [257]. For a recent review about the current state and future directions of ISM, see [258].

## 5. Image Processing

### 5.1. Deconvolution

Different sources of noise, out-of-focus light, and optical diffraction laws limit the image resolution in fluorescence microscopy. Besides improvements in the imaging optics, resolution can be enhanced at the software level. Hardware solutions focus on changing the setup of the instrument, for example, CLSM or multiphoton microscopy, while software solutions increase the resolution of the images by a priori knowledge and denoising. Deconvolution reconstructs a “true” image using the point-spread function of the object. Point-spread functions are typically calculated from acquired images of fluorescent beads or Q-dots. Currently, there are both open-source and proprietary software solutions available for performing deconvolution [259,260]. Although they remain computationally demanding, software solutions have received increasing coverage in virology [261,262,263].

### 5.2. Software Based Super-Resolution

Analytical approaches to super-resolution microscopy are a recent development. They stand out for being entirely software-based, and hence are applicable to a wide range of microscopy modalities [264,265,266]. Similar to super-resolution microscopy, software-based super-resolution obtains information from non-overlapping individual fluorophores. However, rather than requiring specific dyes or excitation conditions, software-based super-resolution relies on sampling fluorophore fluctuation information in a temporal fashion. One of the first software algorithms of this kind was super-resolution optical fluctuation imaging (SOFI) [266]. SOFI is reminiscent to STORM processing algorithms, and relies on collecting cumulants of fluctuating fluorophores [267]. Another method termed 3B analysis utilizes Bayesian statistical analyses to obtain the super-resolution information from the temporal domain [268]. 

The so-called super-resolution radial fluctuations (SRRF) method achieves super-resolution by radial-symmetry based higher-order statistical analysis of temporal intensity fluctuations of conventional fluorophores. Impressively, SRRF achieves 60 nm resolution of images obtained with widefield microscopy, and is extendible to other microscopy modalities [264]. Finally, an approach named NanoJ-SQUIRREL allows a significant improvement in resolution in a wide range of super-resolution modalities, including STED or SIM. The open-source software performs quantitative assessments of super-resolution image quality upon processing, thus creating a metric for improving image processing. Noteworthy, NanoJ-SQUIRREL has been successfully applied to reconstruction of lateral bodies, a structural element of vaccinia virus particles [265,269].

### 5.3. Data Analysis

Image and data analysis is a critical, but somewhat underappreciated aspect of microscopy. Image analysis refers to the extraction of numeric data from a set of images, for example, the number of viral particles bound to the cell, co-localization levels or cell motility features. Quantitative image analysis is an important approach to analyze the cell-to-cell variability in infection [85,270,271,272]. Data analysis refers to the identification of phenotypes of interest and statistical analyses in large datasets. While manual quantification and classification of images is still practiced, manual procedures are prone to confirmation bias, are difficult to standardize and lack scalability. They can result in data misinterpretation and statistically underpowered claims [273,274,275,276]. Automated large-scale experiments have been introduced to biology with the so-called OMICS technologies in the 1990s. Yet, it is still a challenge to standardize automated methods for image and data analysis. Nevertheless, the increased demand for statistically powerful experiments and reproducible analysis pipelines will reward the implementation of standardized approaches.

**Figure 1:**
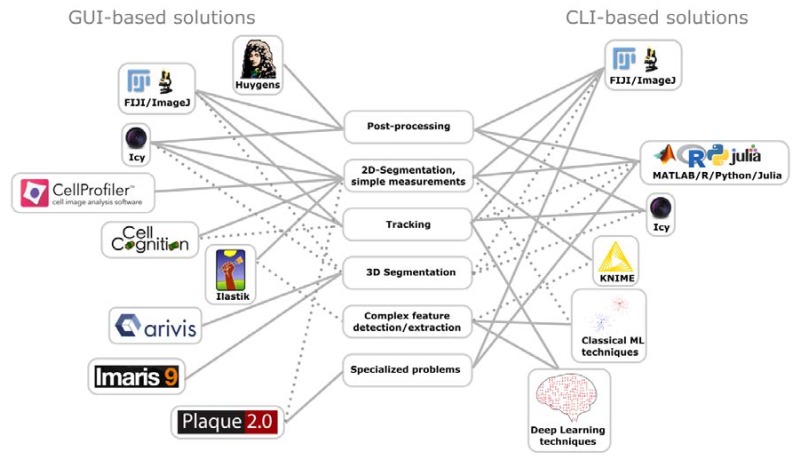
Overview of popular image processing and analyses procedures. Comparison of graphical user interface (GUI) and command line interface (CLI) solutions, which can be used for image post-processing, including denoising, and deconvolution. GUI and CLI are further used for object segmentation in 2D and 3D, particle tracking, for example virions and cells, complex feature detection and extraction by pattern recognition, clustering, multiparametric classification, or inference. GUI and CLI are also used for specialized problems, representing specialized software for assessing particular biological phenotypes. Thick lines denote primary applications of a software/framework for a particular problem. Dashed lines denote secondary applications.

Analysis tools can be broadly generalized into command-line interface (CLI) and graphical user interface (GUI) based tools (Figure 1). Typical programming languages in life sciences for the CLI-based approach are R, Python, and MATLAB. A key advantage of CLIs is flexibility and scalability as well as transparency of the underlying methodology. Drawbacks include a cost for the user to become proficient in at least one programming language for being able to develop analysis pipelines. Proficiency is important, because programming errors can result in misrepresentation of the data. Fortunately, the academic and commercial communities recognize the demand for analysis tools. Today, a wide array of plugins, toolboxes or ready-made analysis software exists. Comprising of either simple to use CLIs or GUIs which offer approachable solutions requiring little to none knowledge of programming.

Most microscope manufacturers provide acquisition software that is capable of performing standard post-processing and image analysis steps. However, the analysis and processing tools provided by dedicated research groups and companies tend to show superior performance. This is partially due to compatibility issues between different commercial solutions, and lower prioritization of software development by the manufacturers.

The best-known tool for academic image processing is perhaps ImageJ. It was originally developed in 1997 and is still maintained by Wayne Rasband [277]. ImageJ is an open-source platform, which allows for image visualization and processing, and incorporates several hundred analysis plugins. A deep strength of ImageJ is the ease with which additional plugins can be generated, modified, and installed. The prime example for this is Fiji, a recursive acronym for “Fiji Is Just ImageJ”. Fiji is an implementation of ImageJ which is expanded by many plugins, as well as an integrated updating system and developer tools [260]. Several plugins perform surprisingly complex tasks at high quality, including 3D stitching [278], generation of super-resolution images from diffraction limited image stacks [264], or lineage tracing [279].

While ImageJ/Fiji provides a powerful and simple to use toolbox, the creation of new plugins or adaption of existing ones can become challenging for people not familiar with “Java”, a general-purpose programming language. Fortunately, there are several open-source programs available, which generate flexible analysis pipelines by combining existing modules in an easy-to-use GUI.

For example, CellProfiler (http://cellprofiler.org/) is an image analysis software that allows the simple generation of automated workflows for high-content imaging [280]. The resulting datasets can be exported as .csv files, and employed for further analysis. A useful tool for data analysis is KNIME (Konstanz Information Miner—https://www.knime.com/). While capable of image analysis KNIME has a stronger focus on data analysis, allowing easy handling and exploration of datasets containing several million datapoints [281]. Similar to ImageJ, it has a framework, which enables simple implementation of additional nodes, strengthening the core functionalities by features, such as Java/R/Python/MATLAB/ImageJ compatibility, machine learning or expanded workflow control. Icy (http://icy.bioimageanalysis.org/) combines both image and data analysis. While slightly less intuitive to use compared to CellProfiler/KNIME, Icy provides great segmentation quality even for noisy images and a similar wide array of functionality and compatibility plugins [282].

Apart from the general solutions for image and data analyses, a number of specific solutions for microbiological questions have been designed. For example, based on the idea of the original plaque assay of Dulbecco [283], an analysis software termed “Plaque2.0” was designed, which allows automated scoring of lesions or fluorescence labeled virus spreading events in high-throughput format, providing more information at lower resource consumption, reduced incubation time, and larger scale than prior procedures [284].

An alternative approach to tackle the amount of data generated by recent advances in biomedical imaging, such as high-throughput time-lapse microscopy, is machine learning (ML). ML refers to a family of computer science methods that allow for automatic learning and then recognizing, classifying, and predicting of patterns in a dataset. In a biomedical imaging context, ML is typically used for relatively simple tasks, such as segmentation, tracking, denoising, and phenotype determination. For example, tools like Ilastik [285] and CellCognition provide easy-to-use and powerful ways to segment and classify of images by reinforced machine learning [286].

Recent advances in computing, specifically in graphical processing units, have enabled efficient implementations of artificial neural networks and deep learning, which vastly outperformed classical ML methods in image recognition problems [287,288]. These methods allow for detection and prediction of highly complex biological phenotypes. Moreover, visualization of these trained networks can help to identify new patterns and features of phenotypes [289,290]. Furthermore, deep neural networks can be used to improve super-resolution processing [291]. Deep learning remains an actively developing field and promises to lead to breakthroughs in biomedicine.

## 6. Emerging Techniques 

### 6.1. Correlative Light and Electron Microscopy

Correlative light and electron microscopy (CLEM) is accurately described by its name. While both methods have long been established, the staining protocols differ significantly and tend to destroy either the epitopes, the fluorophores, or the ultrastructures [292]. CLEM has been used to study structural changes in AdV infected cells in the 1960s [293], and it became clear that the results differed significantly depending on preservation and fixation methods. In 1973, Tokuyasu published an embedding technique, which allowed the correlative staining while maintaining the ultrastructure of the sample [294]. Still, for several decades CLEM has remained impractical to use for most virological applications.

In recent years, multiple improvements were made in hardware, such as the integration of fluorescence into electron microscopes [295], improved sample preparation techniques [296,297], the development of improved probes [298], and better processing software complemented the advances [299,300]. Modern implementations of CLEM use complex protocols that generate tomographic CLEM images of virus infected cells [301,302], allow live cell fluorescence imaging [303], or combine it with super-resolution optical microscopy [304]. In recent years CLEM has been used to study various aspects of the viral replication cycle, such as entry [305,306], replication [307,308] or egress [309]. For a more extensive review regarding CLEM in virus–host interactions, see [310].

### 6.2. Digital Holographic Microscopy

Digital holographic microscopy (DHM) allows for non-invasive, label-free 3D imaging of objects situated on a transparent support. The object, for example a live cell, is examined by a low energy laser beam, which is split into two beams, one passing through the object and the other one through an object-free zone. The interaction of the beams yields an interferogram, and upon transformation, a 3D image of the object [24,311].

This concept has recently been used to construct a microscope, which measures refractive indices in biological samples and generates tomographic 3D images up to 150 nm axial and 75 nm lateral resolution [312]. Advantages of this system include label-free, non-invasive, and fast 3D image acquisition, which is compatible with live imaging. A commercial microscope has become available in 2015 under the brand name 3D Cell Explorer. So far, it was mainly used for whole cell analysis [313,314]. The combination of non-invasive, non-phototoxic, label-free cell tomography at high temporal and spatial resolution now allow the measurements of physical properties of cells that have so far been hard to achieve. These properties include the refractive indices and refractive gradients, and promise new insights into the cell biology of virus infections.

## 7. Conclusions

The direct visualization of host–pathogen interactions by optical microscopy has not been feasible for a long time due to physical limitations in sensitivity, resolution, and lack of appropriate imaging devices and labels. In the last decade, tremendous advances were made in key areas of light microscopy. These advances included novel labeling strategies with ultra-high sensitivity, such as bDNA-FISH and click-chemistry labeling of nucleic acids, lipids, and proteins, as well as improved dyes and photon detectors allowing effective detection of individual particles and molecules in live cells and chemically fixed cells. A vast array of super-resolution imaging modalities is enabling the localization and separation of structures at resolutions that were considered impossible for centuries. The combination of improved assays, microscopes, and image and data analyses now allows one to conduct imaging experiments with nanometer resolutions, 3D live object sampling, and scoring time-resolved cell and infection phenotypes in high-throughput mode.

Advances in imaging and data analysis will continue to enhance our understanding of host–pathogen interactions, and impact on immunology, epidemiology, and public health. Nevertheless, and despite all these advances, we must not forget to emphasize the key importance of finding and raising the fundamental questions and hypotheses in virus research. How do things work the way we see them? For example, how do replicating viruses give rise to phase separated zones in the cytoplasm or the nucleus, as observed by fluorescence microscopy [315]? We pose that the combination of informed hypotheses, imaging and thorough data analyses will advance our understanding of biology and virus infections in unexpected and exciting ways.

## Figures and Tables

**Table 1:** Primary applications of light microscopy systems in virus infections of cell cultures and tissues.

	Live Acquisition	Long Term Acquisition	3D Acquisition	High-Throughput	Super-Resolution	Deep Tissue	FRET Compatible
Widefield	+	+		+			
CLSM	+		(+)				+
SDCM	+	+	+	+			+
2-photon			+		+	+	+
Airyscan	+	+	+	+	+		+
Lightsheet	+	+	+			+	
STED	(+)				+		
PALM/STORM					+		
SIM	(+)		+		+		
iSIM	+	+	+	(+)	+		(+)
DHM	+	+	+		+		

Well suited applications are denoted by +, non-suited by blank fields, and partially suited by (+). Note that specialized systems, such as 3D-SIM [19,20], or Live-STED [21] have been reported but have not been widely used in infection biology and therefore are not considered here. Abbreviations: CLSM—Confocal Laser Scanning Microscope, DHM—Digital Holographic Microscope, FRET—Fluorescence/Förster Resonance Energy Transfer, iSIM—Instant Structured Illumination Microscopy, PALM—Photo-Activated Localization Microscopy, SDCM—Spinning-Disk Confocal Microscope, SIM—Structured Illumination Microscopy, STED—Stimulated Emission Depletion, STORM—Stochastic Optical Reconstruction Microscopy.

**Table 2:** Classification of imaging infrastructure by “ease of use”, “maintenance”, “data size”, “quantifiable data output”, and “post-processing requirements”. “Simple” instruments can be operated after one day of training. “Advanced” instruments have more complex modalities and parameter settings, and require additional training. “Expert” instruments require multiple weeks of training, including experimental set up, appropriate controls and calibrations. Star (*) denotes that the user-friendliness is strongly dependent on the particular setup. “Maintenance” estimates whether the standard procedures including hardware management can be carried out by a trained user, a specialist, or an engineer, for example in a dedicated imaging facility. “Data size” refers to image files of a typical experiment, ranging from “small” (megabyte range) to “very large” (hundreds of gigabytes). Note that time course or high-throughput experiments significantly increase data size. “Quantifiable data output” denotes if a linear ratio of fluorescence excitation to emission is obtained, which can be used for intensity comparisons between different experimental conditions. “Post-processing” indicates whether images can be directly used for analysis, or if they require additional steps such as alignment, averaging or reconstruction.

	Ease of Use	Maintenance	File Sizes	Quantifiable	Postprocessing
Widefield	Simple	User	Small	Yes	No
CLSM	Simple	Specialist	Moderate	Yes	No
SDCM	Simple	Specialist	Moderate	Yes	No
2-photon	Expert	Engineer	Moderate	Yes	No
Airyscan	Simple	Engineer	Moderate	Yes	Yes
Lightsheet	Advanced	Specialist	Very large	Yes	Yes
STED	Advanced	Specialist	Small	Yes	No
PALM/STORM	Simple–Expert *	Specialist	Very large	No	Yes
SIM	Simple–Expert *	Engineer	Moderate	No	Yes
iSIM	Simple	Engineer	Moderate	Yes	No
DHM	Simple	User	Large	Yes	Yes

Abbreviations: CLSM—Confocal Laser Scanning Microscope, DHM—Digital Holographic Microscope, iSIM—instant Structured Illumination Microscopy, PALM—Photo-Activated Localization Microscopy, SDCM—Spinning Disk Confocal Microscope, SIM—Structured Illumination Microscopy, STED—Stimulated Emission Depletion, STORM—Stochastic Optical Reconstruction Microscopy.

## Abbreviations

**Microscopy**

CARS	Coherent Anti-Stokes Raman Spectroscopy
CLEM	Correlative Light and Electron Microscopy
CLSM	Confocal Laser Scanning Microscope
DHM	Digital Holographic Microscope
dSTORM	Direct Stochastic Optical Reconstruction Microscopy
EM	Electron Microscope
ExM	Expansion Microscopy
FRAP	Fluorescence Recovery after Photobleaching
FLIM	Fluorescence-Lifetime Imaging Microscopy
FRET	Fluorescence/Förster Resonance Energy Transfer
iSIM	Instant Structured Illumination Microscopy
ISM	Image Scanning Microscopy
OPFOS	Orthogonal Plane Fluorescence Optical Sectioning
PAINT	Points Accumulation for Imaging in Nanoscale Topography
PALM	Photo-Activated Localization Microscopy
QE	Quantum Efficiency
SDCM	Spinning-Disk Confocal Microscope
SIM	Structured Illumination Microscopy
SNR	Signal-to-Noise Ratio
SPIM	Selective Plane Illumination Microscopy
STED	Stimulated Emission Depletion
STORM	Stochastic Optical Reconstruction Microscopy
TIRF	Total Internal Reflection Fluorescence

**Viruses**

AAV	Adeno-Associated Virus
AdV	Adenovirus
HBV	Hepatitis B Virus
HCV	Hepatitis C Virus
HIV	Human Immune-Deficiency Virus
HPV	Human Papilloma Virus
HSV	Herpes Simplex Virus
IAV	Influenza A Virus
RSV	Respiratory Syncytial Virus
SV	Simian Virus
TMV	Tobacco Mosaic Virus
VACV	Vaccinia Virus

**Labeling**

bDNA	Branched DNA
ExM	Expansion Microscopy
(e)GFP	(Enhanced) Green Fluorescent Protein
(F)ISH	(Fluorescence) *In Situ* Hybridization
FUCCI	Fluorescent Ubiquitination-based Cell Cycle Indicator
IF	Immunofluorescence
Q-Dots	Quantum Dots
SiR	Silicon-Rhodamine
smFISH	Single Molecule FISH
TRITC	Tetramethylrhodamine

**Analysis**

CLI	Command-Line Interface
GUI	Graphical User Interface
ML	Machine Learning
SOFI	Super-resolution Optical Fluctuation Imaging
SRRF	Super-Resolution Radial Fluctuations
